# Dynamic left ventricular outflow tract obstruction induced by intra-aortic balloon pump in patient with angioedema

**DOI:** 10.1186/s13089-025-00426-4

**Published:** 2025-04-07

**Authors:** Konstantin Yastrebov, Gregory Cranney

**Affiliations:** 1 University of New South Wales, Kensington, Sydney, NSW 2033 Australia; 2 Prince of Wales Hospital, Barker Street, Sydney, NSW 2031 Australia; 3 The University of Sydney, Camperdown, Sydney, NSW 2050 Australia

## Abstract

**Background:**

Intra-aortic balloon pump is used for temporary mechanical support of failing left ventricle. It works by reducing the arterial afterload during ventricular systole to reduce myocardial work and increasing diastolic proximal aortic pressure to improve coronary perfusion. Rarely, intra-aortic balloon pump (IABP) can become the cause of severe haemodynamic compromise, causing dynamic left ventricular outflow tract obstruction.

**Case presentation:**

An 88-yo man presented with angiotensin converting enzyme inhibitor (ACEI) - induced angioedema. He received steroids and adrenaline, but progressed to the respiratory arrest, requiring emergency awake fiberoptic intubation and mechanical ventilation. Echocardiography revealed catecholamine-induced reversed Takotsubo cardiomyopathy. The patient suffered asystolic cardiac arrest on arrival to intensive care unit (ICU), requiring cardiopulmonary resuscitation (CPR). Bradycardia and hypotension were treated with atrial pacing and (IABP). Icatibant was administered for angioedema. After several hours of haemodynamic stability, severe hypotension returned. Bedside echocardiographic diagnosis of recovery from Takotsubo and new development of IABP-induced dynamic left ventricular outflow tract obstruction (DLVOTO) was made. Stopping IABP resulted in rapid haemodynamic recovery. Repeated doses of Icatibant were needed. The patient survived and returned to independent living.

**Conclusions:**

Immediate echocardiographic recognition of iatrogenic DLVOTO caused by IABP allows discontinuation of IABP support as a life-saving intervention. Dynamic application of spectral Doppler with changes in IABP settings is required for correct diagnosis.

## Background

Excessive production of bradykinin on the background of C1 inhibitor deficiency in acquired angioedema results in increased microvascular permeability and fluid extravasation into tissues, including the larynx. ACEIs are widely used in clinical practice and may cause angioedema in 0.1–0.7% of cases [1] at any time due to the decreased degradation of bradykinin [2, 3]. 30 mg subcutaneous Icatibant competitively blocks B2 bradykinin receptors, although single dose may be insufficient [4]. Our patient required 3 doses to achieve complete resolution of angioedema. Treatment of ACEI-induced angioedema with adrenaline, steroids or antihistamine agents is ineffective and can cause iatrogenic complications, including Takotsubo.

DLVOTO is an intermittent significant narrowing of the LVOT causing partial obstruction for systemic blood flow from the left ventricle during systole. Anatomical predispositions for DLVOTO include interventricular septal hypertrophy, excessively elongated anterior mitral leaflet and reduced aorto-mitral angle. Physiological factors include hyperdynamic LV, reduced arterial afterload and hypovolemia. High flow velocity across narrowed left ventricular outflow tract (LVOT) causes systolic anterior motion of the mitral valve, degrees of LVOT obstruction, failure of MV leaflets coaptation and mitral incompetence. Takotsubo is characterized by hyperdynamic basal segments and has been associated with the development of DLVOTO [5], diagnosed by bedside echocardiography. Inodilators, including Levosimendan, increase myocardial contractility including basal left ventricular segments while reducing LV afterload– both effects contributing to the exacerbation existing DLVOTO.

Reverse Takotsubo is described as an acute myocardial ischaemia without severe underlying large coronary artery disease with characteristic hypokinetic basal left ventricular segments and hyperkinetic apex (thus the “reverse” to the traditionally described Takotsubo). Its causative list is presumed to resemble classic Takotsubo syndrome with similar rates of resulting cardiogenic shock. Its incidents represent 1–23% of all diagnosed Takotsubo syndromes [6]. 

IABP offers mechanical reduction in systolic LV afterload being used to support patients with cardiogenic shock [7]. Initially successful support of a failing LV in our patient with IABP, subsequently induced iatrogenic DLVOTO, when basal LV segments became hyperdynamic with resolution of reverse Takotsubo. Rapid echocardiographic diagnosis of DLVOTO is essential for terminating harmful therapies, preventing further progress of shock and pulmonary oedema. Six international reports of IABP-associated DLVOTO were found, none following resolution of reversed Takotsubo [8–13]. 

## Case report

An 88-year-old man presented with unilateral right-side tongue swelling, progressing over few hours to the dysphagia and dyspnoea. He denied any trauma, allergies, or consumption of new foods. His past medical history included hyperlipidaemia and arterial hypertension treated with 10 mg Ramipril daily for 10 years. He lived independently mobilizing without aid.

Intramuscular and nebulized adrenaline and intravenous dexamethasone were administered in the emergency department (ED), failing to abort progressive swelling of the airways. Nasal endoscopy by ENT confirmed severe oedema of the tongue and the larynx. Mechanical ventilation was initiated following fiberoptic intubation with the patient developing peri-intubation respiratory arrest. Chest X-ray and transthoracic echocardiography (TTE) performed by the cardiology advanced trainee in the ED confirmed negative pressure pulmonary oedema complicated by acute reverse Takotsubo cardiomyopathy. The patient suffered a brief bradycardic episode, followed by asystolic cardiac arrest on ICU arrival, requiring 7 min of CPR, followed by return to spontaneous circulation. Coronary angiography was performed and was deemed unremarkable. Sinus bradycardia and an unstable hemodynamic state demanded concurrent insertion of atrial pacing wire and intra-aortic balloon pump (IABP). The choice of mechanical circulatory support by IABP was dictated by the desire to avoid the use of inotropes in presence of acute left ventricular failure and arterial hypotension. Stable pacing rhythm and excellent augmentation of pressures by IABP provided stabilization of global perfusion. TTE demonstrated persistent left ventricular failure due to catecholamine induced reverse Takotsubo syndrome (good apical left ventricular (LV) contraction with hypokinesis of basal and mid-LV segments). Mitral valve (MV) had normal function. Levosimendan infusion was started. A diagnosis of angiotensin-converting enzyme inhibitor (ACEI)-induced angioedema was confirmed by the immunologist. The first dose of Icatibant was administered.

**Fig. 1:**
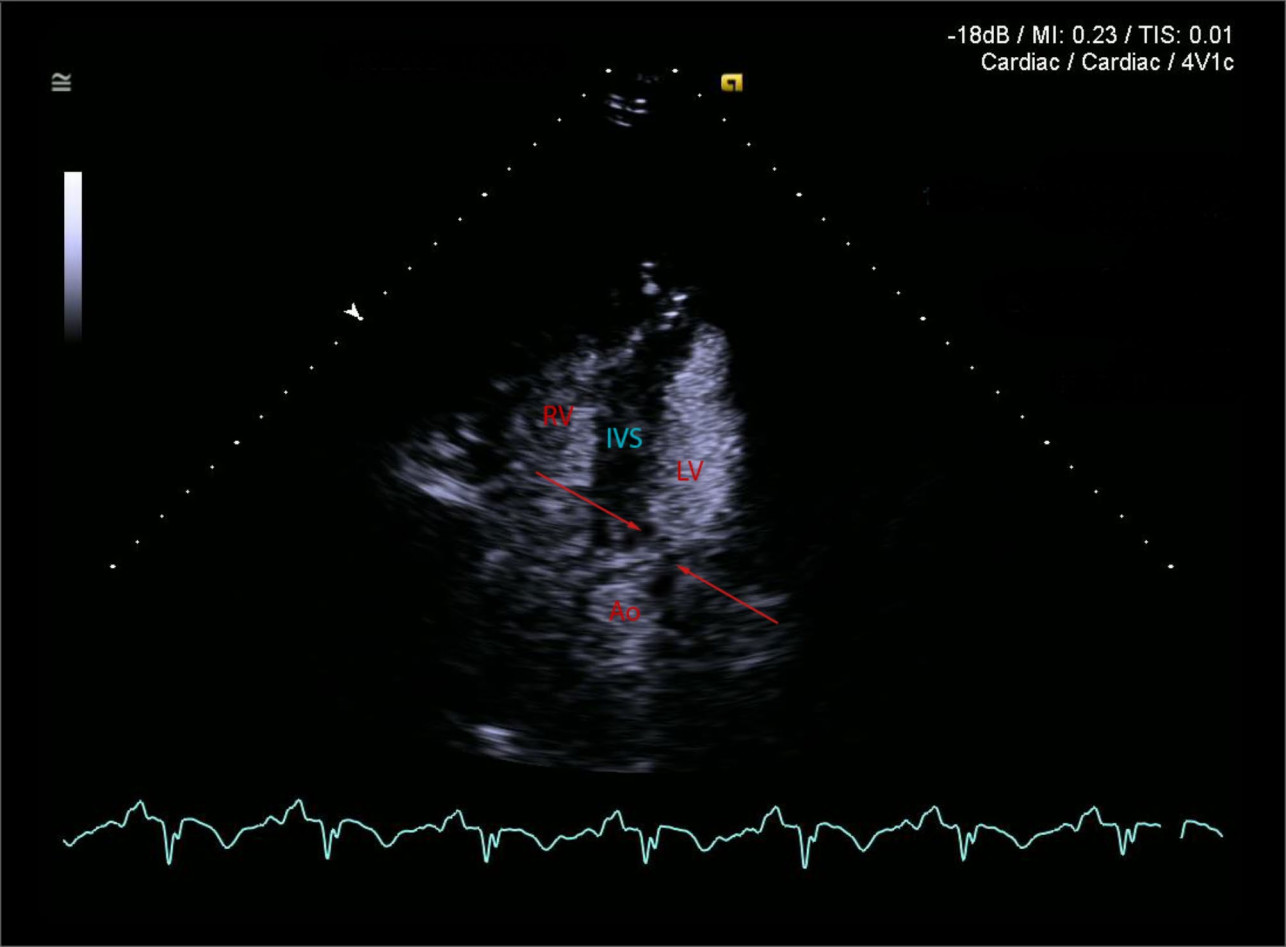
Systolic frame of the apical 5-chamber TTE view with administered ultrasound enhancing agent and low mechanical index settings of the scanner. Narrowing of the LVOT is noted (red arrows)

**Fig. 2:**
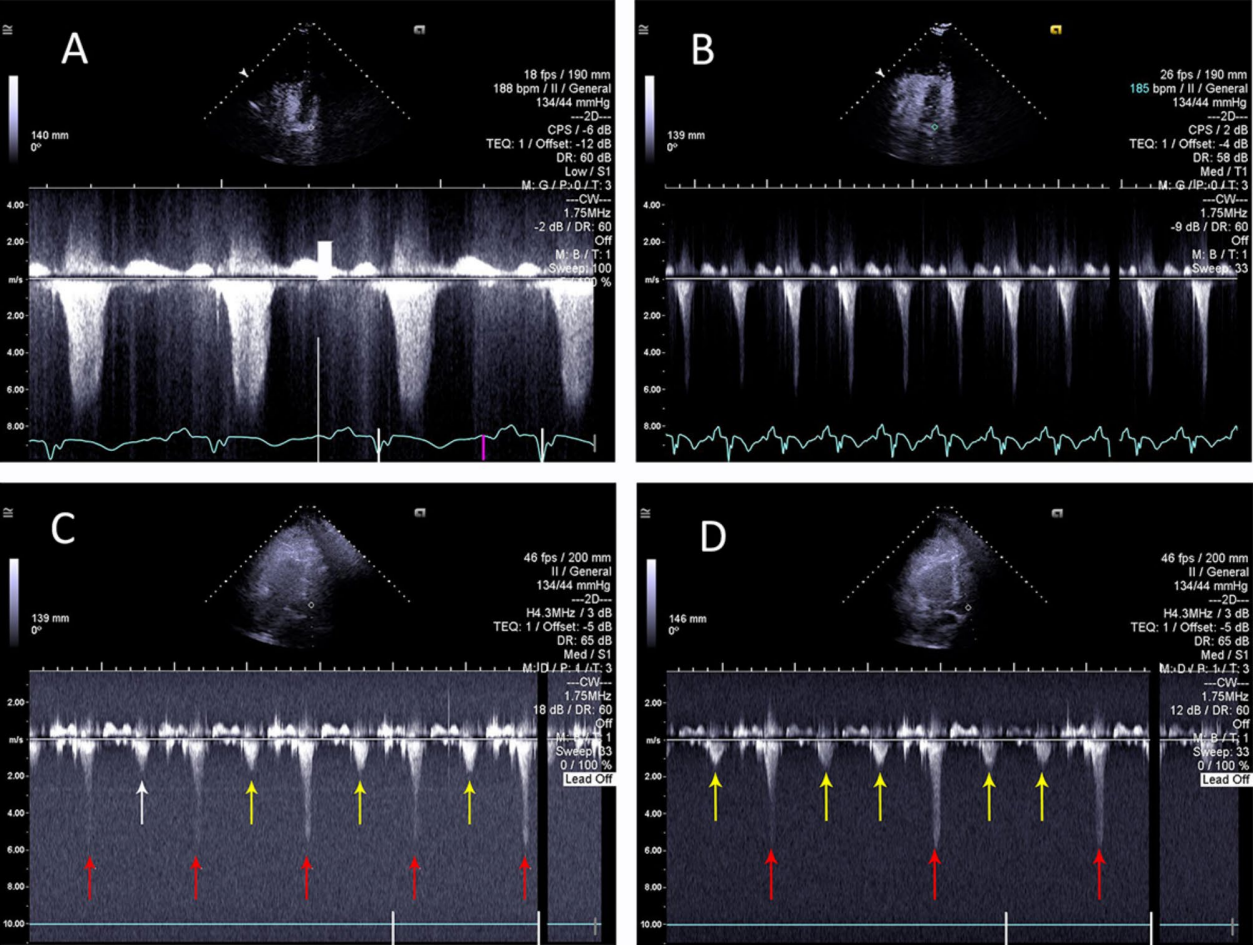
Panel **A**: Continuous Wave Doppler signal sampled from mitral regurgitant jet during IABP support with the ratio 1:1. It demonstrated a maximum LV/LA pressure gradient of 235 mmHg. Note the MR CW signal is late-systolic Panel **B**: Continuous Wave Doppler signal sampled from LVOT during IABP support with the ratio 1:1. It demonstrated a maximum LV/aorta pressure gradient of 125 mmHg. Note the typical dagger-shaped Doppler profile compared to the more rounded shape of the MR jet in panel **A** Panel **C**: Continuous Wave Doppler signal sampled from LVOT during IABP support with the ratio 1:2. Red arrows indicate amended by IABP cardiac cycles with elevated maximum pressure gradient across LVOT. Yellow arrows indicate unamended by IABP every second cardiac cycle with normal pressure gradient across LVOT Panel **D**: Continuous Wave Doppler signal sampled from LVOT during IABP support with the ratio 1:3. Yellow arrows indicate two unamended by IABP cardiac cycles with normal pressure gradient across LVOT for each one amended by IABP cycles (red arrows)

Patient became haemodynamically unstable again next morning with systolic blood pressure drop to 60 mmHg. Poor TTE windows and ongoing mechanical ventilatory support resulted in suboptimal echocardiographic imaging. Remaining significant upper airways oedema precluded the use of transoesophageal echocardiography. TTE with ultrasound enhancing agent, performed by the intensive care specialist with advanced echocardiographic training, demonstrated hyperdynamic LV with complete recovery of all segments, indicating resolution of reverse Takotsubo. Narrowing of the LVOT was noted on 2D imaging (Fig. 1). Color Doppler revealed a new posteriorly directed mitral regurgitant jet and flow acceleration within the left ventricular outflow tract (LVOT), confirmed by severe aliasing of Pulse Wave Doppler. Continuous Wave Doppler (CWD) had a typical dagger-shaped systolic flow in LVOT (maximum pressure gradient 125 mmHg– Fig. 2 Panel B) and more round-shaped mitral regurgitant flow (maximum pressure gradient 235 mmHg– Fig. 2 Panel A). Changing IABP augmentation ratio from 1:1 to 1:2 (Fig. 2 Panel C) to 1:3 (Fig. 2 Panel D) resulted in reduction of LVOT velocities from 5.5 m/sec to 1.5 m/sec during non-augmented cardiac cycles, remaining high during augmentation. Progressive analysis of LVOT Doppler velocities during amendments of IABP augmentation ratios, confirmed causation of iatrogenic circulatory compromise and the diagnosis of IABP-induced dynamic left ventricular outflow tract obstruction (DLVOTO) was made. Stopping IABP resulted in rapid haemodynamic stabilization. IABP was removed and Levosimendan was discontinued. TTE next day demonstrated normal biventricular systolic function, absence of DLVOTO and completely resolved mitral incompetence.

C3, C4 and C1-estherase inhibitor levels were normal, but persistent angioedema required two more doses of Icatibant. Airways swelling was finally resolved, and the patient was extubated on day 6. He underwent rehabilitation and was discharged home to independent living.

The lessons from our case are centered around prompt multidisciplinary approach to the treatment of life-threatening angioedema and DLVOTO. This case demonstrates how the initial beneficial effects of IABP may rapidly cause near-fatal iatrogenic complications, when causing DLVOTO. Frequent echocardiographic reassessment of unstable patients with mechanical circulatory support in ICU is essential for successful outcome. Echocardiography is best performed by the intensive care or cardiology trained specialist who has sufficient knowledge, training and the authority to temporarily augment IABP settings to allow dynamic echocardiographic assessment.

## Conclusions

- Life-threatening ACEI induced angioedema can develop at any stage of therapy and requires sometimes repeated administration of Icatibant. Adrenaline should not be used to treat ACEI-induced angioedema, except in cases where the diagnosis of anaphylaxis is not excluded.
- Recovery of reversed Takotsubo cardiomyopathy is associated with hyperdynamic basal LV segments and can provoke DLVOTO when coupled with other anatomical, physiological or iatrogenic predisposing factors. Adrenaline and inodilators should be avoided or used with extreme caution. Echocardiographic monitoring is essential when treating patients with Takotsubo, who may develop DLVOTO.
- When assessing the LVOT gradient by Doppler, it is important to identify both the MR jet and LVOT jet. The velocity of the MR jet will always be greater and Doppler profile more rounded, while the LVOT CWD signal with be “dagger shaped”.
- Mechanical circulatory support with IABP can aid in the treatment of cardiogenic shock but may rapidly become detrimental due to the reduction in systolic arterial afterload in patients with predispositions to SAM and DLVOTO. The clinician’s awareness of this rare iatrogenic complication and access to a rapid bedside dynamic echocardiographic diagnosis with augmented settings of IABP is essential for preventing potentially lethal consequences. The presence of cardiology or intensive care specialists with advanced training in echocardiography is required for immediate diagnosis and appropriate rapid intervention. Expertise using ultrasound enhancing agents is valuable.

## Data Availability

Data sharing is not applicable to this article as no new data was created or analyzed in this study.

## Declarations

### Authors’ information

Konstantin Yastrebov: Senior Staff Specialist in Intensive Care, Prince of Wales Hospital, Sydney, Australia. Conjoined Professor, UNSW and Adjunct Professor, USYD. Gregory Cranney: Head of Cardiology and Senior Staff Specialist in Cardiology, Prince of Wales Hospital.

## Abbreviations

LV: Left Ventricle
LVOT: Left ventricular outflow tract
DLVOTO: Dynamic left ventricular outflow tract obstruction
TTE: Transthoracic echocardiography
IABP: Intra-aortic balloon pump
MV: Mitral valve
SAM: Systolic anterior motion of the mitral valve
CWD: Continuous wave Doppler
ACEI: Angiotensin-converting enzyme inhibitors
